# Investigation of the efficacy and safety of wild- type and triple-gene knockout pig RBC transfusions in nonhuman primates

**DOI:** 10.3389/fimmu.2024.1418249

**Published:** 2024-06-27

**Authors:** Juhye Roh, Jeong Ho Hwang, Sangkeun Park, Haneulnari Lee, Eun Mi Park, Hye Won Lee, Ju Young Lee, Joohyun Shim, Kimyung Choi, Hee Jung Kang

**Affiliations:** ^1^ Department of Laboratory Medicine, Hallym University College of Medicine and Hallym University Sacred Heart Hospital, Anyang, Republic of Korea; ^2^ Animal Model Research Group, Jeonbuk Branch Institute, Korea Institute of Toxicology, Jeongeup, Republic of Korea; ^3^ Department of Transgenic Animal Research, Optipharm Inc., Cheongju, Republic of Korea

**Keywords:** porcine red blood cells, xenotransfusion, transfusion, nonhuman primates, genetically modified pigs

## Abstract

**Introduction:**

Decreasing rates of blood donation and close margins between blood supply and demand pose challenges in healthcare. Genetically engineered pig red blood cells (pRBCs) have been explored as alternatives to human RBCs for transfusion, and triple-gene knockout (TKO) modification improves the compatibility of pRBCs with human blood *in vitro*. In this study, we assessed the efficacy and risks of transfusing wild-type (WT)- and TKO-pRBCs into nonhuman primates (NHPs).

**Methods:**

Blood from O-type WT and TKO pigs was processed to produce pRBCs for transfusion, which were transfused or not into NHPs (n=4 per group: WT, TKO, and control) after 25% total blood volume withdrawal: their biological responses were compared. Hematological, biochemical, and immunological parameters were measured before, immediately after, and at intervals following transfusion. Two months later, a second transfusion was performed in three NHPs of the transfusion group.

**Results:**

Transfusion of both WT- and TKO-pRBCs significantly improved RBC counts, hematocrit, and hemoglobin levels up to the first day post-transfusion, compared to the controls. The transfusion groups showed instant complement activation and rapid elicitation of anti-pig antibodies, as well as elevated liver enzyme and bilirubin levels post-transfusion. Despite the higher agglutination titers with WT-pRBCs in the pre-transfusion crossmatch, the differences between the WT and TKO groups were not remarkable except for less impairment of liver function in the TKO group. After the second transfusion, more pronounced adverse responses without any hematological gain were observed.

**Conclusions:**

WT- and TKO-pRBC transfusions effectively increased hematologic parameters on the first day, with rapid clearance from circulation thereafter. However, pRBC transfusion triggers strong antibody responses, limiting the benefits of the pRBC transfusion and increasing the risk of adverse reactions.

## 1 Introduction

The steady decline in blood donation rates without a reduction in the number of transfusions has led to a narrowing of blood supply and demand, raising concerns within medical societies (1). To prevent the potential risk of blood shortage, researchers are developing alternative products or technologies to replace human blood for transfusion (2). Porcine red blood cells (pRBCs) are a potential alternative to human RBCs (hRBCs) for transfusion (3). Transfusion of animal blood is not a new concept; it was attempted in the past, and its potential value was recognized in emergencies with acute hemorrhage until the 19^th^ century (4, 5). Since the identification of the ABO blood groups (6), allogeneic hRBCs have become a standard for transfusion. However, owing to recent progress in genetic technologies (7, 8), significant advances have been made in organ and tissue xenotransplantation (9, 10). Transplantation of organs from genetically modified pigs dramatically prolongs the survival of xenografts in non-human primates (NHPs) (11). Therefore, pRBCs from genetically modified pigs have been explored as alternatives to hRBCs (12). Previously, we investigated the compatibility of pRBCs from several genetically modified pigs with human sera *in vitro* and found that triple knockout (TKO)-pRBCs with/without further modifications are comparable to O-type hRBCs (13), where TKO pigs are devoid of expressing major xenoantigens: galactose-α-1,3-galactose (αGal), *N*-glycolylneuraminic acid, and Sd(a) on the surface of pig cells. Next, to assess the feasibility of xenotransfusion *in vivo*, we established a controlled blood loss model in NHPs, in which 25% withdrawal of the total blood volume was appropriate for observing significant hematological changes without consequent lethality of the animal (14). In this study, we investigated the therapeutic impact and adverse reactions of wild-type (WT)- or TKO-pRBC transfusions in NHPs with 25% blood volume loss.

## 2 Materials and methods

### 2.1 Study design

From each WT or TKO pig (Optipharm, Chungcheongbuk-do, Korea) with blood type O, 300 ml of whole blood was collected in a blood bag with acid citrate dextrose solution (Changyoung Medical Co., Chungcheongbuk-do, Korea). The blood in the bag was filtered using a BioR leukocyte depletion filter (Fresenius Kabi AG, Bad Homburg, Germany), washed twice with normal saline, and finally adjusted to its initial hematocrit level using SAG-M additive solution (Changyoung Medical Co.). During the procedure, all blood bags were aseptically connected using a TSCD II sterile tubing welder (Terumo BCT, Inc., Lakewood, CO, USA).

**Figure 1:**
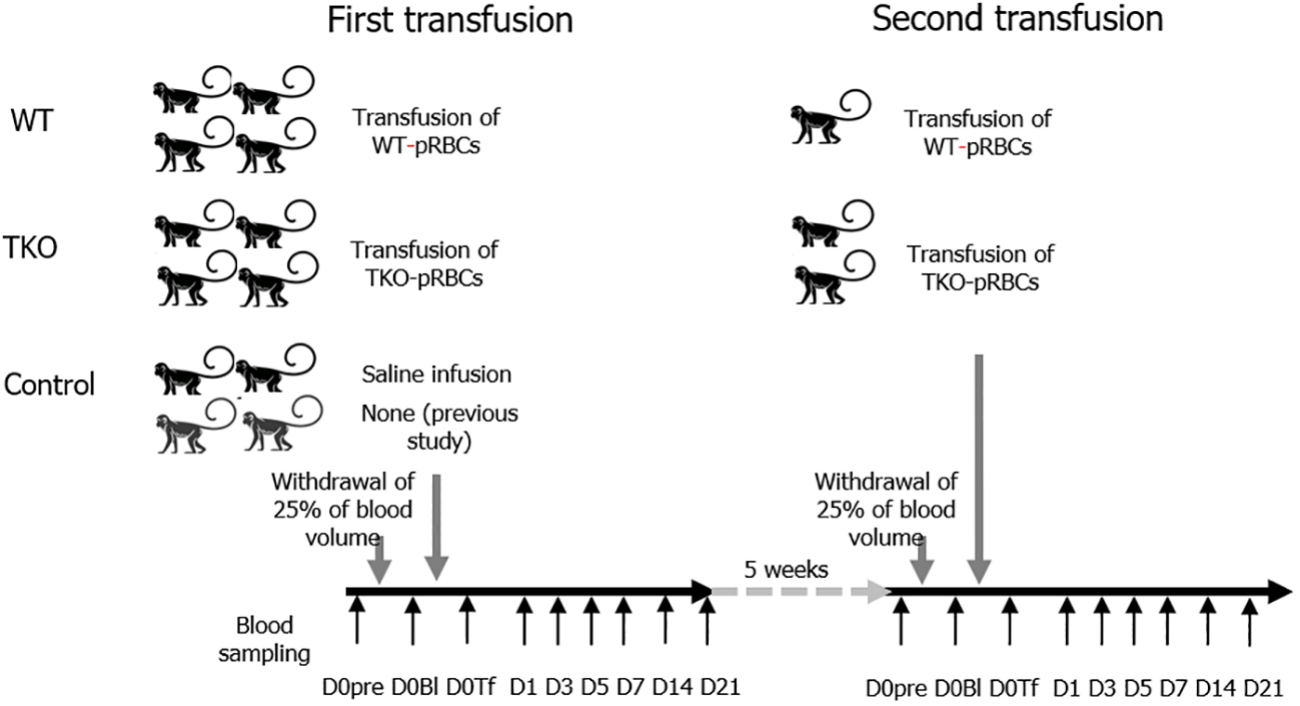
Animal study schema. The 12 nonhuman primates (NHPs) underwent withdrawal of 25% of the total blood volume and were transfused with wild type (WT)- or triple knockout (TKO)-porcine red blood cells (pRBCs) (n=4 each) or infused with normal saline/none (n=4). Blood samples were collected from the NHPs before bleeding (D0pre), immediately after bleeding (D0Bl), and after transfusion/infusion (D0Tf) and on each day (day 1, 3, 5, 7, 14, and 21). Two months later, the second transfusion with pRBCs was conducted in three NHPs (1 WT group and 2 TKO group) following the same protocol. The data and samples of two NHPs from a previous study (14) were included in the control group.

A schematic illustration of the study is shown in **Figure 1**. We used 12 cynomolgus monkeys (*Macaca fascicularis;* Nafovanny, Dong Nai, Vietnam) with authorization from the Korea Institute of Toxicology, Institutional Animal Care and Use Committee (IAC-22–01-0342–0037, IAC-23–01-0389, and IAC-23–01-0302). From all the NHPs, 25% of the total blood volume was removed, and the same amount of WT- or TKO-pRBCs (n=4 per group; male 2, female 2), or saline (n=2; male 1, female 1) as a control was administered to each NHP. The data and leftover samples of the two NHPs with 25% blood withdrawal in our previous study (14) were included in the control group (n=4). Vital signs and general symptoms were observed daily for three weeks from the day of blood withdrawal/transfusion (D0). Blood parameters of the NHPs were analyzed on day 0 before the intervention (D0pre), after blood withdrawal (D0Bl), immediately after the administration of pRBCs or saline/none (D0Tf), and on days 1, 3, 5, 7, 14, and 21 (D1, D3, D5, D7, D14, and D21). For the second transfusion experiment, the monkeys underwent a repeat procedure two months after the first transfusion: two were transfused with TKO-pRBCs and one with WT-pRBCs. General observation and blood sampling were performed using the same protocol.

### 2.2 Measurement of clinical biomarkers

Hematological tests were performed to determine the total white blood cell (WBC) count, total RBC count, hemoglobin (HGB), hematocrit (HCT), mean corpuscular volume (MCV), mean corpuscular hemoglobin concentration (MCHC), mean corpuscular hemoglobin (MCH), platelet count, reticulocyte count (RETA), and WBC differential count. Chemistry tests were performed to measure the levels of glucose (GLU), blood urea nitrogen (BUN), creatinine (CREA), total protein (TP), albumin (ALB), albumin/globulin (A/G) ratio, total cholesterol (TCHO), triglycerides (TG), phospholipids (PL), aspartate aminotransferase (AST), alanine aminotransferase (ALT), alkaline phosphatase (ALP), gamma-glutamyl transpeptidase (GGT), iron, total iron-binding capacity (TIBC), total bilirubin (TBIL), creatine phosphokinase (CK), calcium (Ca), inorganic phosphorus (IP), sodium, potassium, chloride, transferrin saturation (TS), and unsaturated iron-binding capacity (UIBC). Using a cytometric bead array human inflammatory cytokine kit (BD Biosciences, San Diego, CA, USA), we measured plasma levels of interleukin (IL)-1β, IL-6, IL-8, IL-10, IL-12p70, and tumor necrosis factor through flow cytometry. Interferon (IFN)-γ, IL-1α, IL-15, and monocyte chemoattractant protein-1 (MCP-1) levels were measured via enzyme-linked immunosorbent assay (ELISA) using monkey IFN-γ ELISAPRO (MABTECH, Stockholm, Sweden), monkey IL-1α ELISA, monkey IL-15 ELISA (Cusabio, Wuhan, China), and monkey MCP1 ELISA kits (Abcam, Cambridge, UK), respectively. Levels of complement activation fragments C3a and C4a were measured using ELISA with human C3a and C4a ELISA kits (BD Biosciences) and factor Bb was measured with a Bb plus EIA kit (Quidel, San Diego, CA, USA); ELISA and flow cytometry were performed in duplicate.

### 2.3 Measurement of anti-pig antibodies

Plasma concentrations of pig cell-specific immunoglobulin (Ig) M and IgG antibodies were determined by flow cytometry, as described previously (15), using α1,3- galactosyltransferase gene knockout (GTKO) porcine peripheral blood mononuclear cells (pPBMCs) as targeting cells. Briefly, each 1 × 10^5^ GTKO-pPBMCs suspension was incubated in 50 µL of 10% monkey serum or pig serum (as a negative control) diluted in phosphate-buffered saline containing 1% human serum albumin and 33 mM EDTA at 4°C for 1 h and then stained with fluorescein-conjugated F(ab)2 fragments of rabbit immunoglobulins specific for human IgG or IgM (Dako, Santa Clara, CA, USA). Bound pig cell-specific antibodies were measured by flow cytometry, and the levels were expressed as the net mean fluorescence intensity (MFI) after subtracting the MFI of the negative control. Levels of anti-αGal IgG/IgM antibodies and anti-porcine albumin (pALB) IgG antibodies were measured by ELISA, as described previously (16). The levels of anti-αGal IgG/IgM antibodies were titrated using calibrators and expressed in artificial unit (AU)/ml, while the level of anti-pALB was measured using 1/100 diluted samples and expressed as an absorbance of ELISA. Each measurement using ELISA or flow cytometry was performed in duplicate. Crossmatching between monkey serum and pRBCs (WT- or TKO-pRBCs) was performed using the agglutination method with an ID-gel card system (Bio-Rad Laboratories, Inc., Hercules, CA, USA), following the manufacturer’s instructions. Samples with positive agglutination results were titrated using the method described above.

### 2.4 Statistical analysis

Statistical analyses were performed using MedCalc software (version 22.0; Ostend, Belgium). For comparison between groups, the data were transformed into ratios or differences (Δ) based on the results at the D0pre and plotted against each time point (14). The area under the curve (AUC) was compared using one-way analysis of variance. Comparisons between the three groups at each time point were conducted using the Kruskal–Wallis (K-W) test, and in cases where statistical significance was found, differences between each pair were further analyzed using the Mann–Whitney U (M-W) test. The differences in transfusion-related changes between the first and second transfusions were analyzed in the same way. The data were transformed into ratios or differences based on the result of each D0pre, and the differences between the first and second transfusions in the AUCs and the levels at each time point were analyzed using the M-W test. Statistical significance was set at *P* < 0.05.

## 3 Results

### 3.1 Changes in the levels of hematological parameters after pRBC transfusion

**Figure 2:**
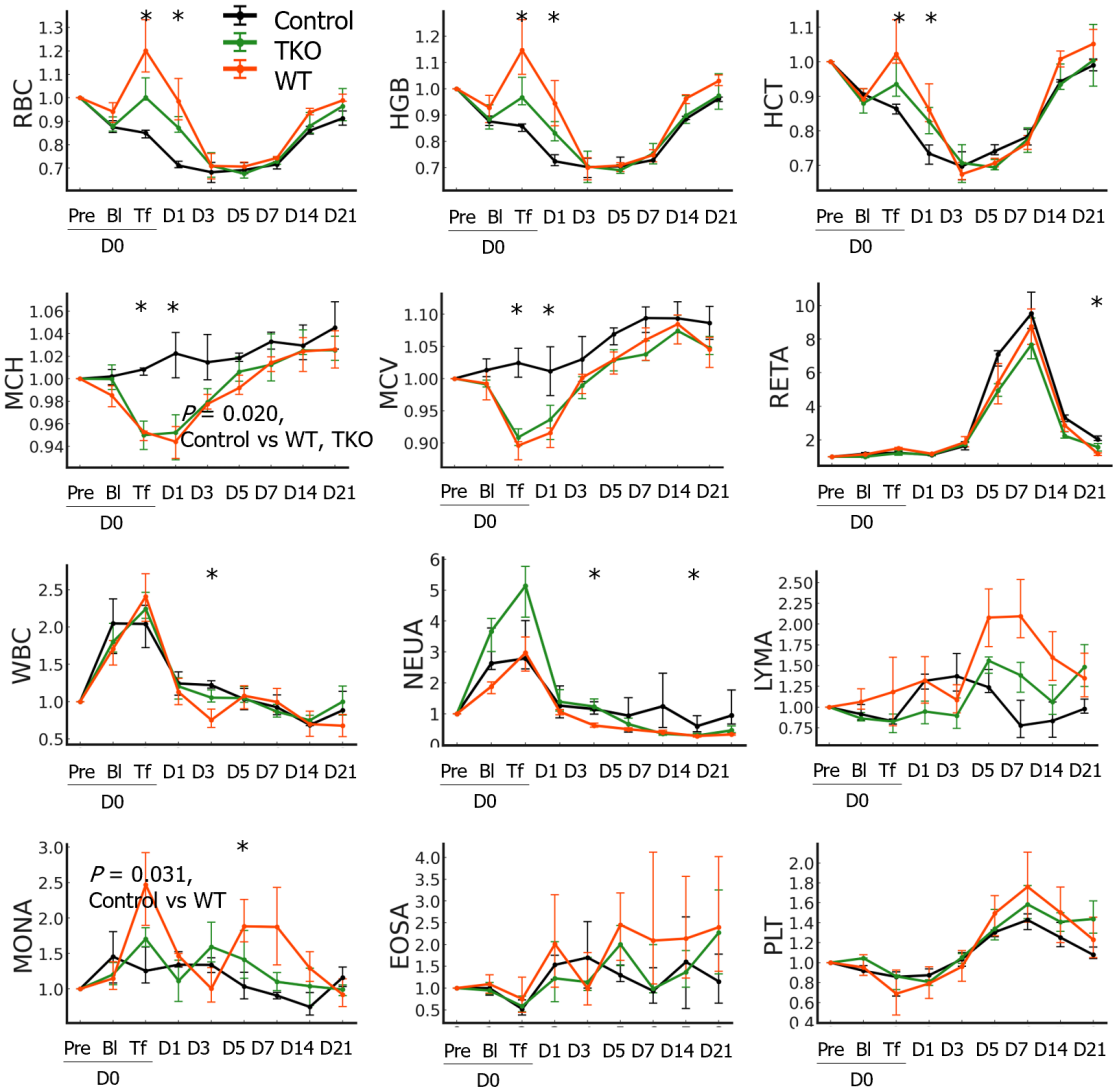
Hematological changes in the nonhuman primates following wild type (WT)- or triple knockout (TKO)-porcine red blood cell (pRBC) transfusion and normal saline infusion (control) (n=4 each). The median ratios to baseline levels with interquartile ranges were plotted. The areas under the curve were compared between the three groups and the *P* values of the groups are expressed on the panel if the difference between groups was significant. The ratio values at each time point were compared between the three groups using the Kruskal–Wallis (K-W) test and significant *P* values are marked (*) on the panel. RBC, red blood cell; HGB, hemoglobin; HCT, hematocrit; MCH, mean corpuscular hemoglobin; MCV, mean corpuscular volume; RETA, reticulocyte; WBC, white blood cell; NEUA, neutrophils; LYMA, lymphocyte; MONA, monocyte; EOSA, eosinophil; PLT, platelet; * *P* < 0.05.

The hematological characteristics of the pRBCs prepared in this study are presented in **Table 1**. The ratios of hematological parameters at each time point compared to those at baseline (D0pre) in the NHPs are shown in **Figure 2**. The AUCs were not significantly different between the three groups in most parameters except for the MCH and monocyte count: the AUC of MCH ratio was significantly greater in the control than in the transfusion groups (*P*=0.020) and that of monocyte count was greater in the WT group than in the control group (*P*=0.031). The changes in RBC count, HGB, and HCT ratios were similar; these values decreased after blood withdrawal (D0Bl) but increased immediately after transfusion (D0Tf) in the transfusion groups, but did not change in the control group, and then decreased, reaching a nadir on D3, and gradually recovered in all groups. The ratio values on D0Tf and D1 were significantly higher in the transfusion groups than in the control group and did not differ between the WT and TKO groups (**Figure 2** and **Supplementary Table 1**). However, the differences between D1 and D3 in RBC count, HGB, and HCT ratio values were significantly greater in WT group than in TKO group (*P*=0.0294, 0.0298, and 0.0298, respectively), suggesting a faster decrease in these values. MCV and MCH ratios changed in a similar pattern: a drop immediately after transfusion, a nadir on D0Tf or D1, and gradual increases in both transfusion groups, whereas there was little change in the controls. RETA ratios started to increase after blood withdrawal and peaked on D7, with no differences among the three groups. The ratio values of WBC and neutrophil counts increased immediately after blood withdrawal and returned to basal levels, without any difference among the three groups; however, monocyte count ratios intermittently increased during the study period only in the transfusion groups, and the value on D7 in the WT group was significantly higher than that in the control group.

**Table 1:** Hematological characteristics of the washed porcine red blood cells.

Parameters	Mean	SD
RBC (×10^6^/µL)	7.2	1.6
Hemoglobin (g/dL)	13.9	3.1
Hematocrit (%)	45.4	9.6
MCV (fL)	63.0	2.7
MCH (pg)	19.3	1.1
MCHC (g/dL)	30.6	0.6
WBC (×10^3^/µL)	Unmeasurable	Unmeasurable

RBC, red blood cell; MCH, mean corpuscular hemoglobin; MCHC, mean corpuscular hemoglobin concentration; MCV, mean corpuscular volume; SD, standard deviation; WBC, white blood cell.

### 3.2 Cytokine, complement, and antibody responses after transfusion of pRBCs

**Figure 3:**
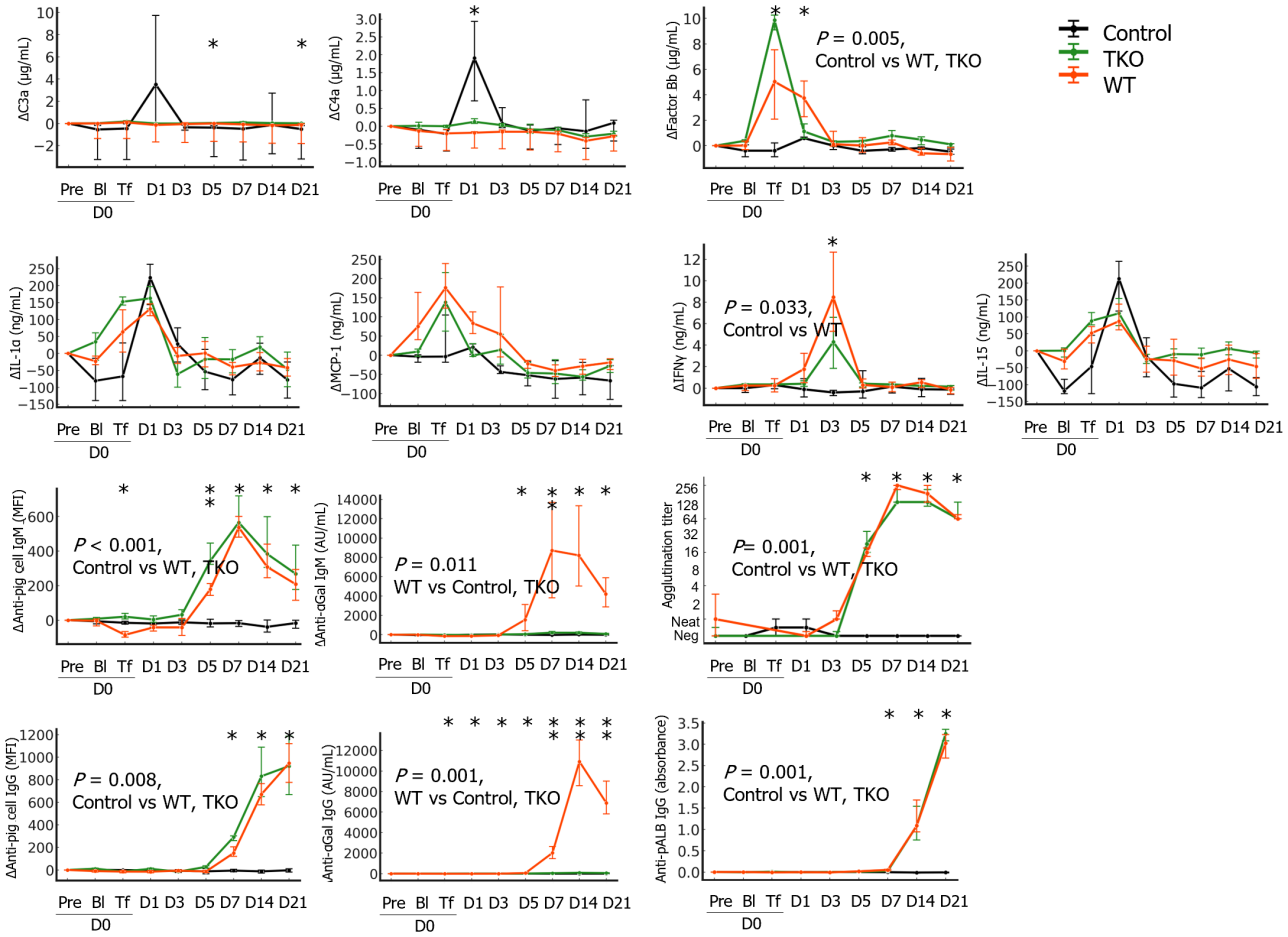
The changes in immunological parameters in the nonhuman primates following wild type (WT)- or triple knockout (TKO)-porcine red blood cell (pRBC) transfusion and normal saline infusion (control group) (n=4 each). The median differences (Δ) in the levels at each point from baseline with interquartile ranges were plotted. The areas under the curve were compared between the three groups and the *P* values of the groups are expressed on the panel if the difference was significant. The values at each time point were compared between the three groups using the Kruskal–Wallis (K-W) test, and significant *P* values are marked (*) on the panel. Anti-pig cell antibodies were measured using α1,3- galactosyltransferase gene knockout porcine peripheral blood mononuclear cells as a target by flow cytometry and levels of anti-αGal IgG/IgM antibodies and anti-porcine albumin (pALB) IgG antibodies were measured by ELISA in αGal and pALB coated wells, respectively. Agglutination with TKO-pRBC was titrated by the ID-gel card system. IL, interleukin; MCP-1, monocyte chemoattractant protein-1; IFN, interferon; *, *P* < 0.05; **, *P* < 0.01.

Among the measured complement activation fragments, Δfactor Bb significantly increased on D0Tf and D1 in the transfusion groups and the Δfactor Bb AUCs of the transfusion groups were significantly greater than those of the control group. In contrast, ΔC3a and ΔC4a intermittently increased during the study period, and the AUCs did not differ between the groups (**Figure 3** and **Supplementary Table 2**). Most cytokines did not change significantly but ΔIL-1α, ΔMCP-1, and ΔIL-15 increased after blood withdrawal/transfusion and normalized within a few days; these changes did not differ between groups. Interestingly, ΔIFN-γ significantly increased on D3 in both transfusion groups, and the AUCs of the WT were significantly greater than those of the control. Transfusion with pRBCs elicits antibody responses against diverse porcine antigens. When GTKO-pPBMCs were used, significant IgM and IgG binding was observed as early as D5 and D7, respectively. When using αGal-coated wells, similar IgM and IgG antibody binding were detected in the samples of the WT group, but not the TKO group. Notably, anti-αGal IgG levels of the WT group initially decreased after transfusion and were lower than those of the TKO group until D3 but significantly increased on D5, suggesting initial antibody adsorption and subsequent elicitation in the WT group (**Supplementary Table 2**). When pALB-coated wells were used, significant IgG antibody binding was observed as early as D7 in both transfusion groups. In the pre-transfusion crossmatch, agglutination titers of the WT group ranged between 1:8 and 1:32 with WT-pRBCs and between negative and 1:8 with TKO-pRBCs, while the titers of the TKO group were between negative (n=3) and 1:2 (n=1) with TKO-pRBCs. Despite variations in agglutination titers in the pre-transfusion crossmatch, the negative pretransfusion crossmatch primates did not demonstrate a different outcome that their higher titer counterparts. Agglutination titers of the serial samples with TKO-pRBCs were compared; the titers initially decreased but started to increase on D3 in both transfusion groups, while the titer did not change in the control group.

### 3.3 Changes in the levels of biochemical parameters after pRBC transfusion

**Figure 4:**
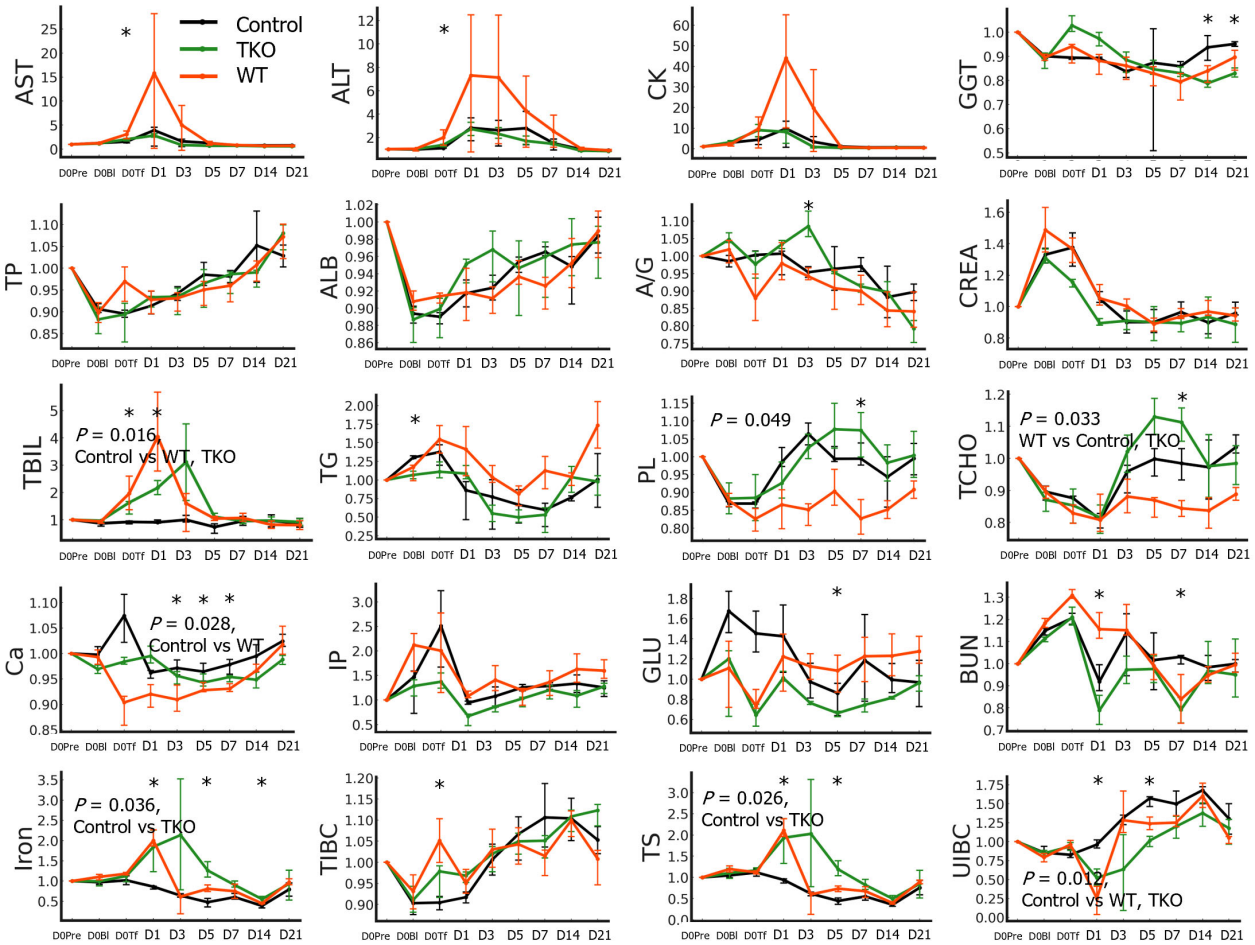
Biochemistry test results of the nonhuman primates following wild type (WT)- or triple knockout (TKO)-porcine red blood cell (pRBC) transfusion and normal saline infusion (control group) (n=4 each). The median ratios to baseline levels with interquartile ranges were plotted. The areas under the curve were compared between the three groups and the *P* values of the groups are expressed on the panel if the difference was significant. The ratio values at each time point were compared between the three groups using the Kruskal–Wallis (K-W) test, and significant *P* values are marked (*) on the panel. AST, aspartate aminotransferase; ALT, alanine aminotransferase; CK, creatine phosphokinase; GGT, gamma glutamyl transpeptidase; TP, total protein; ALB, albumin; A/G, albumin/globulin ratio; CREA, creatinine; TBIL, total bilirubin; TG, triglyceride; PL, phospholipids; TCHO, total cholesterol; Ca, calcium; TIBC, total iron-binding capacity (TIBC); TS, transferrin saturation; UIBC, unsaturated iron-binding capacity. *, *P* < 0.05.

Although the AUCs of AST and ALT ratio values did not differ between the groups, the values of AST and ALT on D0Tf were significantly higher in the WT group than in the control group (**Figure 4** and **Supplementary Table 3**). A portion of the WT group showed tens- or hundred-fold increases in the ratio values of AST, ALT, or CK on D1. The ratio values of TP and ALB initially decreased, started to recover, and normalized over baseline, whereas those of CREA initially increased and normalized under baseline; they did not differ between groups. Both transfusion groups showed an increase in TBIL ratio values, with a peak between D0Tf and D3, and their AUCs were significantly greater than those of the control group. Interestingly, the ratio values of PL and TCHO decreased after transfusion and recovered but the recovery was slower in the WT group with significantly smaller AUCs than those of the other groups, suggesting an alteration of lipid synthesis in the liver. Ca ratio was significantly lower in the WT group, iron and TS ratios higher in the TKO group, and UIBC ratio higher in the control group.

### 3.4 Comparison of the baseline levels of measured parameters between the first and second transfusion experiments

**Figure 5:**
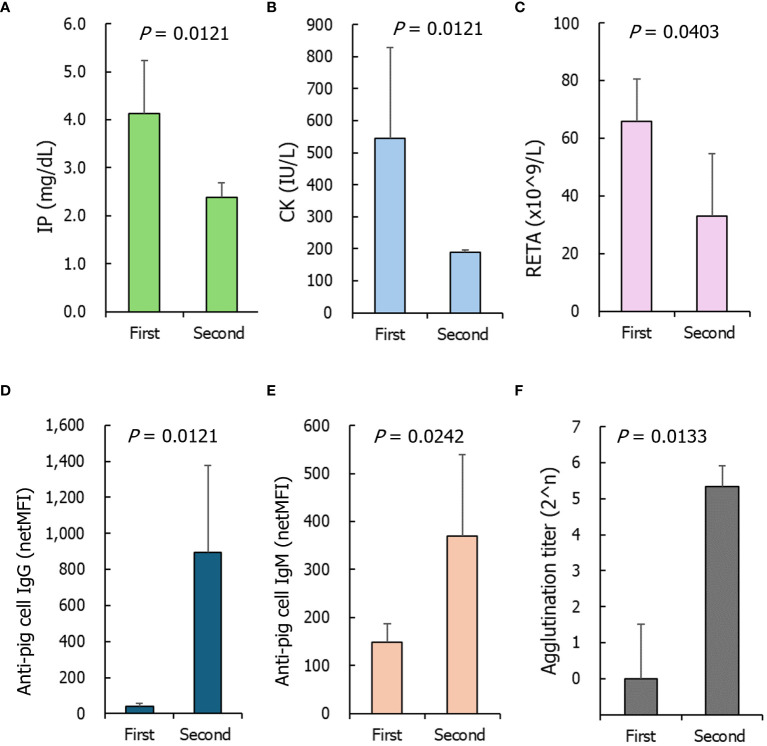
The parameters of nonhuman primates of which baseline levels significantly differed between the first and second transfusions. **(A)** Inorganic phosphorus (IP), **(B)** creatine phosphokinase (CK), **(C)** reticulocyte counts (RETA), (**D**, **E**) anti- α1,3- galactosyltransferase gene knockout porcine peripheral blood mononuclear cells IgG and IgM, **(F)** agglutination titers on triple knockout -porcine red blood cells. The *P* values were obtained using the Mann–Whitney U test. First, n=8; Second, n=3.

When the second transfusion experiments were conducted, the baseline D0pre levels in most parameters did not differ from the D0pre levels of the first transfusions, and only six parameters differed: IP, CK, and RETA on D0pre of the second transfusion were lower than those on D0pre of the first transfusion (**Figures 5A–C**). The levels of IgG/IgM antibodies binding to GTKO-pPBMCs in the D0pre samples from the second transfusion were significantly higher than those from the first transfusion (**Figures 5D, E**). All NHP sera on D0pre of the second transfusion agglutinated TKO-pRBCs at 1:32 or 1:64 titers and were significantly higher than those of the first transfusion samples (**Figure 5F**).

### 3.5 Comparison of biological responses between the first and second transfusion experiments

**Figure 6:**
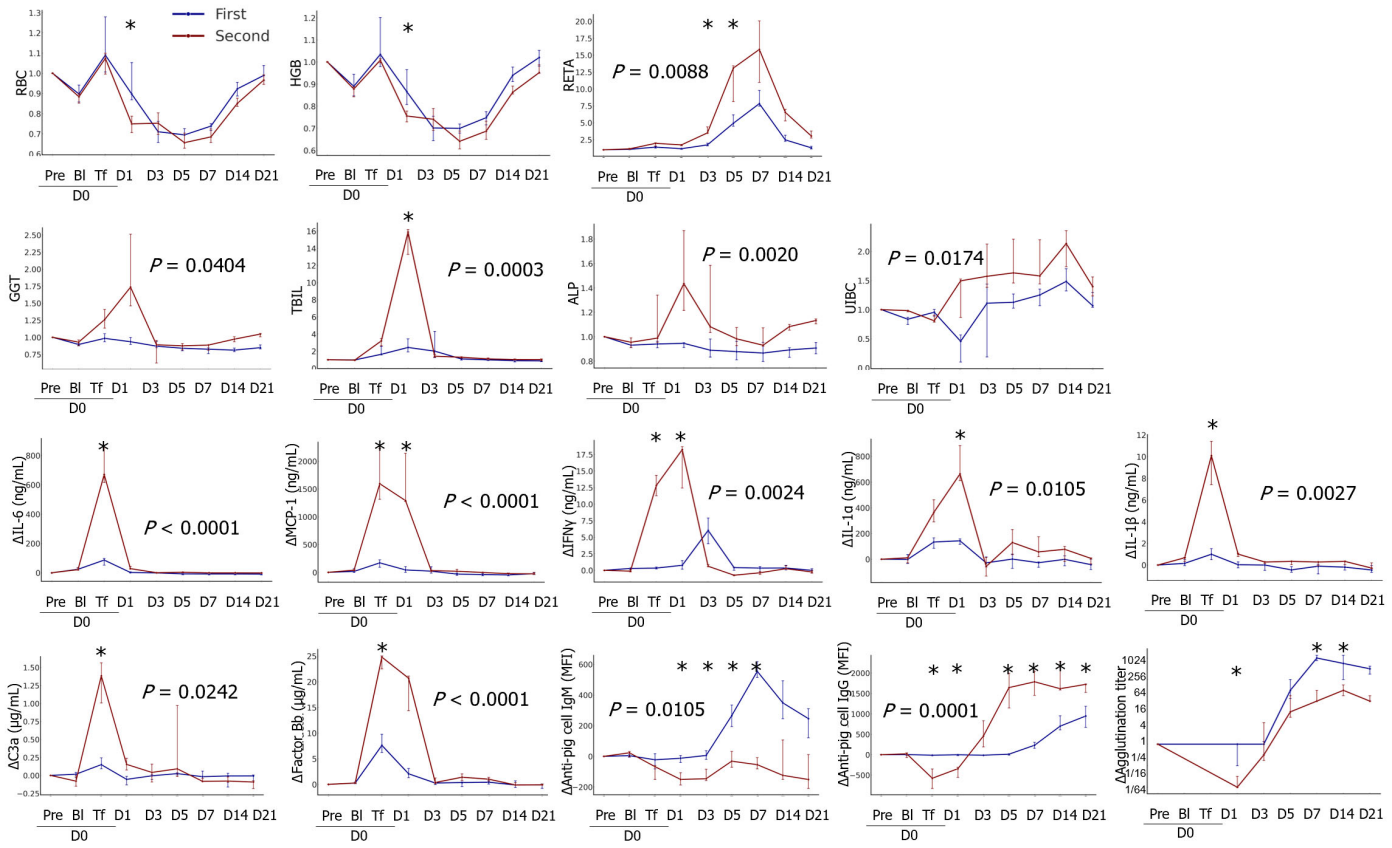
Comparison of transfusion-related changes between the first and second transfusions. The data were transformed into ratios or differences (Δ) based on the results of each pre-transfusion sample before bleeding. The median ratios or differences with interquartile ranges were plotted on the panel. The areas under the curve were compared between groups, and the *P* values are expressed on the panel if the difference was significant. The differences in the levels at each time point between the groups were analyzed using the Mann–Whitney U test, and significant *P* values are marked (*) on the panel (first, n=8; second, n=3). RBC, red blood cell; HGB, hemoglobin; RETA, reticulocyte; GGT, gamma glutamyl transpeptidase; TBIL, total bilirubin; ALP, alkaline phosphatase; IL, interleukin; MCP-1, monocyte chemoattractant protein-1; IFN, interferon. *, *P* < 0.05.

Most adverse reactions to pRBC transfusion were more severe after the second transfusion than after the first transfusion, and the parameters that significantly differed between the first and second transfusions are shown in **Figure 6** and **Supplementary Table 4**. Increases in RBC and HGB ratios on D1 after the first transfusion were abolished on D1 of the second transfusion. The increases in RETA, GGT, TBIL, and ALP ratio values were higher during the second transfusion than during the first transfusion. After the second transfusion, a female patient transfused with TKO-pRBCs showed a sharp increase in CREA levels, suggesting the development of intravascular hemolysis (data not shown). Severe adverse reactions in the second transfusion were prominent in cytokine profiles: IL-6, MCP1, IL-1α, and IL-1β significantly increased after the second transfusion. An increase in the level of IFN-γ appeared earlier, and the level of ΔIFN-γ was higher during the second transfusion compared to the first. More complement activation fragments, C3a and factor Bb, were produced in the second transfusion. The responses of anti-GTKO-pPBMC IgM antibodies after the second transfusion were blunt; however, the IgG responses appeared earlier and were stronger than those after the first transfusion. The increasing tendency in agglutination titers did not differ between the first and second transfusions. However, considering the titer of D0pre, the titers were significantly higher in the second transfusion, reaching 1:2048 compared to those in the first transfusion.

## 4 Discussion

The medical community has voiced concerns about the potential instability of blood supply (17, 18). To address this issue, studies are being conducted to develop alternatives to traditional blood transfusions, including blood substitutes and animal blood (2). Historically, xenogeneic blood transfusions were practiced but abandoned due to the higher risks compared to allogeneic transfusions (3). There is also a lack of modern scientific evaluation of their safety and efficacy. Recently, genetically engineered pigs exhibiting reduced immune rejection in humans have been developed, and our previous study showed that pRBCs from these pigs were comparable to human blood *in vitro* (13). However, considering the complex biological and immunological responses of living subjects, their *in vivo* effectiveness remains unclear. Survival of these pRBCs has been explored in primate models (19, 20), although the pathophysiological changes in transfused primates have not been thoroughly investigated. Therefore, this study introduced transfusions of both WT and genetically modified TKO-pRBCs into NHPs with serious blood loss. Their hematological, biochemical, and immunological responses, along with their overall health, were monitored to assess safety and risks. This study provides the first objective preclinical data on NHPs with pRBC transfusions.

In our previous NHP blood-loss model study (14), we observed a significant increase in WBCs, including neutrophils, regardless of transfusion, likely owing to stress and compensatory mechanisms. The pRBC preparations used leukocyte-depletion filters, resulting in leukocyte counts below measurable limits (**Table 1**). Thus, the increase in WBC and neutrophil counts post-transfusion is unlikely to be caused by leukocytes in the transfused blood. Instead, it is more plausibly attributed to the body’s natural response to blood loss and subsequent transfusion. Furthermore, the initial increase in hematological indicators such as RBC and Hb that was followed by a drop below baseline levels can be attributed to fluid redistribution, which is a normal physiological response to blood loss (14). Over time, erythropoiesis resumes, restoring these indicators to normal levels.

Interestingly, hematological profiles did not significantly differ between WT and TKO groups. This suggests that the fate of the transfused pRBCs in NHPs was similar regardless of the genetic modifications. It is possible that antibodies were rapidly induced against other porcine antigens, leading to the swift phagocytosis of pRBCs. The primary factor responsible for pRBC clearance remains unidentified.

In this study, the hematological benefit after transfusion was evident; both WT and TKO O-type pRBCs existed in the circulation, at least on the first day of transfusion, although they disappeared within three days. Despite stronger positive reactions in the pre-transfusion crossmatch with WT-pRBCs compared to TKO-pRBCs, the overall profiles of HGB, HCT, and RBC did not differ between the WT and TKO groups. Notably, the MCV and MCH in pRBCs are smaller than those in NHPs (14). Thus, decreases in MCH and MCV values after transfusion indirectly reflect the presence of pRBCs in circulation. Similarly, the profiles of proinflammatory cytokine activation did not differ between the transfusion groups. Clinically, ABO-incompatible transplantation is possible unless the agglutination titers are greater than the target range, for example, 1:8 for kidney transplantation (21) or 1:16 for heart transplantation (22). Thus, it is not surprising that transfusion of WT-pRBC with positive agglutination in the pre-transfusion crossmatch was well tolerated in the NHPs and may explain the historical use of animal blood. Nonetheless, the disadvantages of WT-pRBCs over TKO-pRBCs were observed in some findings such as faster disappearance of transfused pRBCs between D1 and D3, increased monocyte counts, and impaired hepatic lipid synthesis after transfusion in the WT group.

Regardless of the genetic modification of pRBCs, NHPs that received pRBC transfusion revealed rapid elicitation of anti-pig antibodies; although the levels of anti-Gal IgM and IgG increased only in the WT group as expected, the levels of anti-pig antibodies including anti-GTKO pPBMC IgM and IgG, anti-pALB IgG, and agglutination titers with TKO-pRBCs increased in both groups without any significant difference. The induction of anti-Gal antibody responses did not interfere with the elicitation of other anti-pig antibodies. Increases in these antibodies were detected as early as D3 for IgM and D5 for IgG. Considering the initial binding of preexisting antibodies to transfused RBCs, antibody elicitation must have been induced earlier than D3. Interestingly, the levels of IFN-γ increased on D3 in the WT group, suggesting higher immunological sensitization.

To enhance pRBC survival, addressing direct phagocytosis and antibody-dependent cell-mediated cytotoxicity (ADCC) is crucial (23). pRBCs lack inhibitory signals to prevent phagocytosis by human macrophages. The interaction between human CD47 and signal-regulatory protein alpha inhibits phagocytosis, suggesting that transgenic expression of human CD47 on pRBCs reduces phagocytosis and prolongs survival. Additionally, brief treatment with the anti-CD154 antibody to inhibit antibody induction and subsequent ADCC could also be beneficial (24).

Severe adverse reactions after transfusion were observed in the NHPs with the second transfusion: faster disappearance of the transfused pRBCs, higher reticulocyte increases, higher levels of liver enzymes and TBIL, and striking increases in pro-inflammatory cytokines. Notably, there were no significant differences in the D0pre samples between the first and second transfusions except for the levels of anti-pig antibodies. This suggests that higher pre-existing antibody levels may explain the severe adverse reactions. Thus, the agglutination titer in the pre-transfusion crossmatch is more important than the qualitative result in predicting the consequences of transfusion. Antibody responses in NHPs with pRBC transfusion were not limited to erythrocyte-specific antigens and extended even to pALB. In this study, we washed pRBCs twice for transfusion and reduced the content of contaminating pALB to a minimum; however, it induced antibody responses in all recipients of transfusion. These findings suggest that further knockout of porcine erythrocyte antigens is unlikely to improve the survival of pRBCs *in vivo*. Instead, because the disappearance of pRBCs within a few days after the first transfusion seems to be mainly mediated by extravascular hemolysis, genetic modifications that interfere with the removal of pRBCs from circulation in the reticuloendothelial system and/or transient immune suppression to inhibit antibody responses would be beneficial for further improvement of pRBC survival.

We expected serious adverse reactions, including lethality, in the WT group; however, surprisingly, no serious adverse reactions were observed in the WT group after the first transfusion. This is possibly because of tolerable agglutination titers, use of O-type pRBCs to avoid ABO incompatibilities, and extensive filtering and washing of pRBCs to reduce post-transfusion allergic reactions. Notably, there were no cases of post-transfusion allergic reactions. We recommend that these measures be implemented as standard procedures for future xenotransfusions. In addition, while we did not perform chest X-rays to observe lung conditions directly, we closely monitored the NHP subjects for signs of acute respiratory distress and hypoxemia post-transfusion. None of the subjects exhibited symptoms of rapid onset hypoxemia or dyspnea, which are characteristic indicators of transfusion-related acute lung injury.

Because of the limited number of experimental animals, we used data and samples from our previous study. In the previous study, fluid resuscitation was not administered to NHPs after blood withdrawal of 25% blood volume, which resulted in a transient hypotensive episode. Thus, in this study, normal saline was administered to the control NHPs to simulate a clinical situation; the rest of the procedure was identical. It is unlikely that this difference would affect the measurement of biological parameters in this study.

This study has some limitations. First, we could not directly measure the lifespan or physiological functions of transfused pRBCs. Second, the pRBC clearance pathway remains unclear. Third, we could not evaluate the contribution of extravascular and intravascular hemolysis to the clearance because of limitations in blood sampling. We can only speculate from increases of TBIL in our results that transfused RBCs seem to be mostly cleared by extravascular hemolysis (phagocytosis by macrophages) and that the contribution of intravascular hemolysis to the pRBC clearance became greater in the second transfusion than that in the first transfusion, which may be attributed to the increase in the levels of anti-pig antibody. The measurement of plasma hemoglobin and haptoglobin levels in NHPs may help evaluate the extent of intravascular hemolysis.

The hematological benefit for the first day of transfusion could be helpful in certain clinical emergencies but is not enough to consider the general use of pRBCs as a substitute for hRBCs. However, this is the first step toward clinical xenotransfusion. In future studies, well-tailored genetic modifications of donor pigs and appropriate preparation of pRBCs may further extend the therapeutic benefits. In addition to hematological improvements, the functional benefits of xenotransfusion in NHPs should be addressed.

## Data availability statement

The original contributions presented in the study are included in the article/**Supplementary Material**, further inquiries can be directed to the corresponding author.

## Ethics statement

The animal study was approved by Korea Institute of Toxicology, Institutional Animal Care and Use Committee. The study was conducted in accordance with the local legislation and institutional requirements.

## Author contributions

JR: Writing – original draft. JH: Funding acquisition, Project administration, Writing – review & editing. SP: Writing – review & editing. HL: Methodology, Writing – review & editing. EP: Methodology, Writing – review & editing. HL: Methodology, Writing – review & editing. JL: Methodology, Writing – review & editing. JS: Writing – review & editing. KC: Writing – review & editing. HK: Funding acquisition, Project administration, Writing – original draft.
