# Supplementary material for: Investigation of the efficacy and safety of wild- type and triple-gene knockout pig RBC transfusions in nonhuman primates
Source: Front Immunol. 2024 Jun 27;15:1418249. doi: 10.3389/fimmu.2024.1418249 (PMC11236543; doi:10.3389/fimmu.2024.1418249)
Supplement: Supplementary file 1 [file Table_1.docx]

Supplementary Material

Supplemental Table 1. Median ratio values and interquartile ranges of hematological parameters that differed significantly among the three groups

|  | Time | Group | Median | Q1 | Q3 | K-W  *P*-value |  | Treatment Pair | M-W U  *P*-value |  |
| --- | --- | --- | --- | --- | --- | --- | --- | --- | --- | --- |
| RBC | D0Tf | Control | 0.85 | 0.83 | 0.86 | 0.0154 | * | Control vs WT-pRBC | 0.0286 | * |
|  |  | TKO-pRBC | 1.00 | 0.99 | 1.08 |  |  | Control vs TKO-pRBC | 0.0286 | * |
|  |  | WT-pRBC | 1.20 | 1.11 | 1.33 |  |  | WT-pRBC vs TKO-pRBC | 0.2000 |  |
|  | D1 | Control | 0.71 | 0.70 | 0.73 | 0.0210 | * | Control vs WT-pRBC | 0.0286 | * |
|  |  | TKO-pRBC | 0.87 | 0.85 | 0.92 |  |  | Control vs TKO-pRBC | 0.0286 | * |
|  |  | WT-pRBC | 0.99 | 0.91 | 1.08 |  |  | WT-pRBC vs TKO-pRBC | 0.4857 |  |
| HGB | D0Tf | Control | 0.86 | 0.84 | 0.87 | 0.0154 | * | Control vs WT-pRBC | 0.0286 | * |
|  |  | TKO-pRBC | 0.97 | 0.94 | 1.04 |  |  | Control vs TKO-pRBC | 0.0286 | * |
|  |  | WT-pRBC | 1.15 | 1.06 | 1.26 |  |  | WT-pRBC vs TKO-pRBC | 0.2000 |  |
|  | D1 | Control | 0.72 | 0.71 | 0.75 | 0.0264 | * | Control vs WT-pRBC | 0.0286 | * |
|  |  | TKO-pRBC | 0.83 | 0.80 | 0.88 |  |  | Control vs TKO-pRBC | 0.0571 |  |
|  |  | WT-pRBC | 0.94 | 0.86 | 1.03 |  |  | WT-pRBC vs TKO-pRBC | 0.3429 |  |
| HCT | D0Tf | Control | 0.86 | 0.85 | 0.88 | 0.0366 | * | Control vs WT-pRBC | 0.0286 | * |
|  |  | TKO-pRBC | 0.94 | 0.90 | 1.01 |  |  | Control vs TKO-pRBC | 0.1143 |  |
|  |  | WT-pRBC | 1.02 | 1.00 | 1.12 |  |  | WT-pRBC vs TKO-pRBC | 0.3429 |  |
|  | D1 | Control | 0.73 | 0.70 | 0.76 | 0.0231 | * | Control vs WT-pRBC | 0.0286 | * |
|  |  | TKO-pRBC | 0.83 | 0.79 | 0.87 |  |  | Control vs TKO-pRBC | 0.0286 | * |
|  |  | WT-pRBC | 0.86 | 0.83 | 0.94 |  |  | WT-pRBC vs TKO-pRBC | 0.6857 |  |
| MCV | D0Tf | Control | 1.02 | 1.00 | 1.05 | 0.0210 | * | Control vs WT-pRBC | 0.0286 | * |
|  |  | TKO-pRBC | 0.91 | 0.90 | 0.92 |  |  | Control vs TKO-pRBC | 0.0286 | * |
|  |  | WT-pRBC | 0.90 | 0.87 | 0.90 |  |  | WT-pRBC vs TKO-pRBC | 0.4857 |  |
|  | D1 | Control | 1.01 | 0.97 | 1.05 | 0.0308 | * | Control vs WT-pRBC | 0.0286 | * |
|  |  | TKO-pRBC | 0.94 | 0.91 | 0.96 |  |  | Control vs TKO-pRBC | 0.0571 |  |
|  |  | WT-pRBC | 0.92 | 0.89 | 0.92 |  |  | WT-pRBC vs TKO-pRBC | 0.4857 |  |
| MCH | D0Tf | Control | 1.01 | 1.00 | 1.01 | 0.0244 | * | Control vs WT-pRBC | 0.0286 | * |
|  |  | TKO-pRBC | 0.95 | 0.94 | 0.96 |  |  | Control vs TKO-pRBC | 0.0286 | * |
|  |  | WT-pRBC | 0.95 | 0.94 | 0.95 |  |  | WT-pRBC vs TKO-pRBC | 0.8857 |  |
|  | D1 | Control | 1.02 | 1.00 | 1.04 | 0.0231 | * | Control vs WT-pRBC | 0.0286 | * |
|  |  | TKO-pRBC | 0.95 | 0.93 | 0.97 |  |  | Control vs TKO-pRBC | 0.0286 | * |
|  |  | WT-pRBC | 0.94 | 0.93 | 0.96 |  |  | WT-pRBC vs TKO-pRBC | 0.6857 |  |
| MCHC | D0Tf | Control | 0.99 | 0.97 | 1.00 | 0.0463 | * | Control vs WT-pRBC | 0.0814 |  |
|  |  | TKO-pRBC | 1.05 | 1.03 | 1.06 |  |  | Control vs TKO-pRBC | 0.0286 | * |
|  |  | WT-pRBC | 1.06 | 1.05 | 1.09 |  |  | WT-pRBC vs TKO-pRBC | 0.6857 |  |
| WBC | D3 | Control | 1.22 | 1.18 | 1.28 | 0.0366 | * | Control vs WT-pRBC | 0.0286 | * |
|  |  | TKO-pRBC | 1.05 | 0.99 | 1.16 |  |  | Control vs TKO-pRBC | 0.3429 |  |
|  |  | WT-pRBC | 0.76 | 0.66 | 0.90 |  |  | WT-pRBC vs TKO-pRBC | 0.1143 |  |
| NEUA | D3 | Control | 1.15 | 0.99 | 1.39 | 0.0231 | * | Control vs WT-pRBC | 0.0286 | * |
|  |  | TKO-pRBC | 1.23 | 1.09 | 1.48 |  |  | Control vs TKO-pRBC | 0.6857 |  |
|  |  | WT-pRBC | 0.62 | 0.57 | 0.70 |  |  | WT-pRBC vs TKO-pRBC | 0.0286 | * |
|  | D14 | Control | 0.60 | 0.41 | 0.94 | 0.0435 | * | Control vs WT-pRBC | 0.0286 | * |
|  |  | TKO-pRBC | 0.30 | 0.28 | 0.37 |  |  | Control vs TKO-pRBC | 0.1143 |  |
|  |  | WT-pRBC | 0.28 | 0.25 | 0.29 |  |  | WT-pRBC vs TKO-pRBC | 0.4857 |  |
| MONA | D7 | Control | 0.91 | 0.86 | 0.95 | 0.0173 | * | Control vs WT-pRBC | 0.0286 | * |
|  |  | TKO-pRBC | 1.10 | 0.97 | 1.23 |  |  | Control vs TKO-pRBC | 0.1143 |  |
|  |  | WT-pRBC | 1.87 | 1.34 | 2.43 |  |  | WT-pRBC vs TKO-pRBC | 0.0571 |  |
| RETA | D21 | Control | 2.03 | 1.95 | 2.23 | 0.0388 | * | Control vs WT-pRBC | 0.0286 | * |
|  |  | TKO-pRBC | 1.56 | 1.34 | 1.79 |  |  | Control vs TKO-pRBC | 0.2000 |  |
|  |  | WT-pRBC | 1.17 | 1.07 | 1.23 |  |  | WT-pRBC vs TKO-pRBC | 0.2000 |  |

RBC, red blood cell; HGB, hemoglobin; HCT, hematocrit; MCH, mean corpuscular hemoglobin; MCV, mean corpuscular volume; RETA, reticulocyte; WBC, white blood cell; NEUA, neutrophils; LYMA, lymphocyte; MONA, monocyte; EOSA, eosinophil; PLT, platelet; K-W, the Kruskal–Wallis test; M-W U, the Mann–Whitney U test; *, *P* < 0.05.

Supplemental Table 2. Median difference values and interquartile ranges of immunological parameters that differed significantly among the three groups

| Parameter | | Time | Group | Median | Q1 | Q3 | K-W  *P*-value |  | Treatment Pair | M-W U  *P*-value |  |
| --- | --- | --- | --- | --- | --- | --- | --- | --- | --- | --- | --- |
| C3a | | D5 | Control | -0.38 | -3.00 | -0.19 | 0.0488 | * | Control vs WT-pRBC | 0.2000 |  |
|  |  |  | TKO-pRBC | 0.04 | 0.02 | 0.06 |  |  | Control vs TKO-pRBC | 0.0286 | * |
|  |  |  | WT-pRBC | 0.00 | -1.62 | 0.03 |  |  | WT-pRBC vs TKO-pRBC | 0.3429 |  |
|  |  | D21 | Control | -0.51 | -3.20 | -0.21 | 0.0210 | * | Control vs WT-pRBC | 0.4857 |  |
|  |  |  | TKO-pRBC | 0.01 | 0.00 | 0.02 |  |  | Control vs TKO-pRBC | 0.0286 | * |
|  |  |  | WT-pRBC | -0.14 | -1.80 | -0.03 |  |  | WT-pRBC vs TKO-pRBC | 0.0286 | * |
| C4a | | D1 | Control | 1.91 | 0.71 | 2.94 | 0.0154 | * | Control vs WT-pRBC | 0.0286 | * |
|  |  |  | TKO-pRBC | 0.12 | 0.08 | 0.21 |  |  | Control vs TKO-pRBC | 0.2000 |  |
|  |  |  | WT-pRBC | -0.18 | -0.61 | -0.16 |  |  | WT-pRBC vs TKO-pRBC | 0.0286 | * |
| Factor Bb | | D0Tf | Control | -0.39 | -0.87 | 0.23 | 0.0173 | * | Control vs WT-pRBC | 0.0571 |  |
|  |  |  | TKO-pRBC | 9.87 | 9.10 | 10.27 |  |  | Control vs TKO-pRBC | 0.0286 | * |
|  |  |  | WT-pRBC | 5.03 | 2.08 | 7.54 |  |  | WT-pRBC vs TKO-pRBC | 0.1143 |  |
|  |  | D1 | Control | 0.59 | 0.53 | 0.66 | 0.0264 | * | Control vs WT-pRBC | 0.0286 | * |
|  |  |  | TKO-pRBC | 1.12 | 0.56 | 1.72 |  |  | Control vs TKO-pRBC | 0.3429 |  |
|  |  |  | WT-pRBC | 3.74 | 2.28 | 5.08 |  |  | WT-pRBC vs TKO-pRBC | 0.0571 |  |
| IFNγ | | D3 | Control | -0.41 | -0.70 | -0.21 | 0.0183 | * | Control vs WT-pRBC | 0.0286 | * |
|  |  |  | TKO-pRBC | 4.31 | 1.85 | 6.60 |  |  | Control vs TKO-pRBC | 0.0286 | * |
|  |  |  | WT-pRBC | 8.46 | 5.29 | 12.67 |  |  | WT-pRBC vs TKO-pRBC | 0.3429 |  |
| Anti-pig cell IgM | | D0Tf | Control | -15.3 | -22.9 | -6.4 | 0.0173 | * | Control vs WT-pRBC | 0.0571 |  |
|  |  |  | TKO-pRBC | 20.0 | 6.4 | 38.8 |  |  | Control vs TKO-pRBC | 0.1143 |  |
|  |  |  | WT-pRBC | -85.3 | -98.3 | -65.6 |  |  | WT-pRBC vs TKO-pRBC | 0.0286 | * |
|  |  | D5 | Control | -18.8 | -38.8 | 4.6 | 0.0073 | ** | Control vs WT-pRBC | 0.0286 | * |
|  |  |  | TKO-pRBC | 343.0 | 322.4 | 445.6 |  |  | Control vs TKO-pRBC | 0.0286 | * |
|  |  |  | WT-pRBC | 179.0 | 143.9 | 211.6 |  |  | WT-pRBC vs TKO-pRBC | 0.0286 | * |
|  |  | D7 | Control | -17.3 | -35.6 | -2.9 | 0.0210 | * | Control vs WT-pRBC | 0.0286 | * |
|  |  |  | TKO-pRBC | 565.5 | 537.6 | 720.0 |  |  | Control vs TKO-pRBC | 0.0286 | * |
|  |  |  | WT-pRBC | 537.3 | 480.0 | 600.8 |  |  | WT-pRBC vs TKO-pRBC | 0.4857 |  |
|  |  | D14 | Control | -38.8 | -69.0 | 0.4 | 0.0244 | * | Control vs WT-pRBC | 0.0286 | * |
|  |  |  | TKO-pRBC | 383.3 | 305.0 | 599.1 |  |  | Control vs TKO-pRBC | 0.0286 | * |
|  |  |  | WT-pRBC | 308.3 | 245.4 | 440.8 |  |  | WT-pRBC vs TKO-pRBC | 0.8857 |  |
|  |  | D21 | Control | -17.0 | -46.4 | -0.1 | 0.0210 | * | Control vs WT-pRBC | 0.0286 | * |
|  |  |  | TKO-pRBC | 268.5 | 177.5 | 434.1 |  |  | Control vs TKO-pRBC | 0.0286 | * |
|  |  |  | WT-pRBC | 208.3 | 115.1 | 293.9 |  |  | WT-pRBC vs TKO-pRBC | 0.4857 |  |
| Anti-αGal IgM | | D5 | Control | -10.9 | -42.5 | 31.7 | 0.0210 | * | Control vs WT-pRBC | 0.0286 | * |
|  |  |  | TKO-pRBC | 56.6 | 21.2 | 86.1 |  |  | Control vs TKO-pRBC | 0.4857 |  |
|  |  |  | WT-pRBC | 1554.1 | 410.8 | 3127.8 |  |  | WT-pRBC vs TKO-pRBC | 0.0286 | * |
|  |  | D7 | Control | -32.5 | -61.3 | 7.5 | 0.0073 | ** | Control vs WT-pRBC | 0.0286 | * |
|  |  |  | TKO-pRBC | 210.7 | 159.1 | 323.2 |  |  | Control vs TKO-pRBC | 0.0286 | * |
|  |  |  | WT-pRBC | 8712.1 | 3803.0 | 13707.6 |  |  | WT-pRBC vs TKO-pRBC | 0.0286 | * |
|  |  | D14 | Control | 8.5 | -33.5 | 46.2 | 0.0154 | * | Control vs WT-pRBC | 0.0286 | * |
|  |  |  | TKO-pRBC | 204.7 | 95.6 | 282.5 |  |  | Control vs TKO-pRBC | 0.2000 |  |
|  |  |  | WT-pRBC | 8205.6 | 5039.3 | 13334.0 |  |  | WT-pRBC vs TKO-pRBC | 0.0286 | * |
|  |  | D21 | Control | -18.0 | -35.1 | 16.6 | 0.0154 | * | Control vs WT-pRBC | 0.0286 | * |
|  |  |  | TKO-pRBC | 82.1 | 43.7 | 126.2 |  |  | Control vs TKO-pRBC | 0.2000 |  |
|  |  |  | WT-pRBC | 4202.7 | 2877.2 | 5889.9 |  |  | WT-pRBC vs TKO-pRBC | 0.0286 | * |
| Anti-pig cell IgG | D7 | Control | -5.8 | -16.6 | 6.4 | 0.0183 | * | Control vs WT-pRBC | 0.0286 | * |  |
|  |  | TKO-pRBC | 291.8 | 260.3 | 301.4 |  |  | Control vs TKO-pRBC | 0.0286 | * |  |
|  |  | WT-pRBC | 150.0 | 121.9 | 204.8 |  |  | WT-pRBC vs TKO-pRBC | 0.3429 |  |  |
|  | D14 | Control | -12.8 | -27.1 | 0.0 | 0.0210 | * | Control vs WT-pRBC | 0.0286 | * |  |
|  |  | TKO-pRBC | 830.8 | 650.1 | 1087.9 |  |  | Control vs TKO-pRBC | 0.0286 | * |  |
|  |  | WT-pRBC | 670.0 | 577.5 | 765.8 |  |  | WT-pRBC vs TKO-pRBC | 0.4857 |  |  |
|  | D21 | Control | -3.0 | -18.6 | 10.1 | 0.0249 | * | Control vs WT-pRBC | 0.0286 | * |  |
|  |  | TKO-pRBC | 921.0 | 667.9 | 1187.3 |  |  | Control vs TKO-pRBC | 0.0286 | * |  |
|  |  | WT-pRBC | 947.0 | 775.8 | 1119.9 |  |  | WT-pRBC vs TKO-pRBC | 1.0000 |  |  |
| Anti-αGal IgG | D0Tf | Control | -6.9 | -15.8 | -5.0 | 0.0435 | * | Control vs WT-pRBC | 0.4857 |  |  |
|  |  | TKO-pRBC | 0.1 | -2.0 | 2.2 |  |  | Control vs TKO-pRBC | 0.1143 |  |  |
|  |  | WT-pRBC | -14.3 | -26.0 | -11.6 |  |  | WT-pRBC vs TKO-pRBC | 0.0286 | * |  |
|  | D1 | Control | 1.3 | -2.5 | 8.1 | 0.0373 | * | Control vs WT-pRBC | 0.0571 |  |  |
|  |  | TKO-pRBC | 1.9 | -1.2 | 10.5 |  |  | Control vs TKO-pRBC | 0.8857 |  |  |
|  |  | WT-pRBC | -13.4 | -23.8 | -11.5 |  |  | WT-pRBC vs TKO-pRBC | 0.0286 | * |  |
|  | D3 | Control | 0.0 | -2.8 | 10.1 | 0.0373 | * | Control vs WT-pRBC | 0.0571 |  |  |
|  |  | TKO-pRBC | 0.9 | -0.3 | 8.2 |  |  | Control vs TKO-pRBC | 0.8857 |  |  |
|  |  | WT-pRBC | -10.6 | -17.2 | -7.0 |  |  | WT-pRBC vs TKO-pRBC | 0.0286 | * |  |
|  | D5 | Control | 2.5 | -0.1 | 5.7 | 0.0366 | * | Control vs WT-pRBC | 0.0286 | * |  |
|  |  | TKO-pRBC | 10.4 | 3.0 | 23.7 |  |  | Control vs TKO-pRBC | 0.3429 |  |  |
|  |  | WT-pRBC | 58.5 | 47.7 | 60.4 |  |  | WT-pRBC vs TKO-pRBC | 0.1143 |  |  |
|  | D7 | Control | -4.5 | -8.3 | -0.5 | 0.0073 | ** | Control vs WT-pRBC | 0.0286 | * |  |
|  |  | TKO-pRBC | 46.0 | 31.6 | 84.6 |  |  | Control vs TKO-pRBC | 0.0286 | * |  |
|  |  | WT-pRBC | 2023.4 | 1460.8 | 2634.4 |  |  | WT-pRBC vs TKO-pRBC | 0.0286 | * |  |
|  | D14 | Control | 0.6 | -18.2 | 15.4 | 0.0073 | ** | Control vs WT-pRBC | 0.0286 | * |  |
|  |  | TKO-pRBC | 83.8 | 62.9 | 132.2 |  |  | Control vs TKO-pRBC | 0.0286 | * |  |
|  |  | WT-pRBC | 10923.2 | 8557.7 | 13034.3 |  |  | WT-pRBC vs TKO-pRBC | 0.0286 | * |  |
|  | D21 | Control | -1.8 | -12.8 | 10.6 | 0.0073 | ** | Control vs WT-pRBC | 0.0286 | * |  |
|  |  | TKO-pRBC | 56.9 | 44.8 | 88.4 |  |  | Control vs TKO-pRBC | 0.0286 | * |  |
|  |  | WT-pRBC | 6882.9 | 5822.6 | 9009.0 |  |  | WT-pRBC vs TKO-pRBC | 0.0286 | * |  |
| Agglutination titer (fold difference) | D5 | Control | 0.0 | 0.0 | 0.0 | 0.0140 | * | Control vs WT-pRBC | 0.0211 | * |  |
|  |  | TKO-pRBC | 5.5 | 5.0 | 6.0 |  |  | Control vs TKO-pRBC | 0.0194 | * |  |
|  |  | WT-pRBC | 3.5 | 1.8 | 5.3 |  |  | WT-pRBC vs TKO-pRBC | 0.2881 |  |  |
|  | D7 | Control | 0.0 | 0.0 | 0.0 | 0.0154 | * | Control vs WT-pRBC | 0.0211 | * |  |
|  |  | TKO-pRBC | 8.0 | 8.0 | 8.3 |  |  | Control vs TKO-pRBC | 0.0177 | * |  |
|  |  | WT-pRBC | 7.5 | 6.5 | 8.3 |  |  | WT-pRBC vs TKO-pRBC | 0.4388 |  |  |
|  | D14 | Control | 0.0 | 0.0 | 0.0 | 0.0200 | * | Control vs WT-pRBC | 0.0211 | * |  |
|  |  | TKO-pRBC | 7.5 | 6.8 | 8.3 |  |  | Control vs TKO-pRBC | 0.0211 | * |  |
|  |  | WT-pRBC | 7.0 | 5.8 | 8.3 |  |  | WT-pRBC vs TKO-pRBC | 0.7688 |  |  |
|  | D21 | Control | 0.0 | 0.0 | 0.0 | 0.0147 | * | Control vs WT-pRBC | 0.0211 | * |  |
|  |  | TKO-pRBC | 7.0 | 7.0 | 7.5 |  |  | Control vs TKO-pRBC | 0.0177 | * |  |
|  |  | WT-pRBC | 6.0 | 4.5 | 7.3 |  |  | WT-pRBC vs TKO-pRBC | 0.3562 |  |  |
| Anti-pALB IgG | D7 | Control | -0.003 | -0.009 | 0.000 | 0.0181 | * | Control vs WT-pRBC | 0.0294 | * |  |
|  |  | TKO-pRBC | 0.027 | 0.026 | 0.028 |  |  | Control vs TKO-pRBC | 0.0294 | * |  |
|  |  | WT-pRBC | 0.050 | 0.032 | 0.067 |  |  | WT-pRBC vs TKO-pRBC | 0.3429 |  |  |
|  | D14 | Control | -0.012 | -0.024 | 0.000 | 0.0228 | * | Control vs WT-pRBC | 0.0294 | * |  |
|  |  | TKO-pRBC | 1.086 | 0.751 | 1.541 |  |  | Control vs TKO-pRBC | 0.0294 | * |  |
|  |  | WT-pRBC | 1.088 | 0.941 | 1.691 |  |  | WT-pRBC vs TKO-pRBC | 0.6857 |  |  |
|  | D21 | Control | -0.008 | -0.019 | 0.000 | 0.0181 | * | Control vs WT-pRBC | 0.0294 | * |  |
|  |  | TKO-pRBC | 3.249 | 3.083 | 3.354 |  |  | Control vs TKO-pRBC | 0.0294 | * |  |
|  |  | WT-pRBC | 3.028 | 2.676 | 3.230 |  |  | WT-pRBC vs TKO-pRBC | 0.3429 |  |  |

IL, interleukin; MCP-1, monocyte chemoattractant protein-1; IFN, interferon; K-W, the Kruskal–Wallis test; M-W U, the Mann–Whitney U test; *, *P* < 0.05; **, *P* < 0.01.

.

Supplemental Table 3. Median ratio values and interquartile ranges of biochemical parameters that differed significantly among the three groups

| Parameter | Time | Group | Median | Q1 | Q3 | K-W  *P*-value |  | Treatment Pair | M-W U  *P*-value |  |
| --- | --- | --- | --- | --- | --- | --- | --- | --- | --- | --- |
| AST | D0Tf | Control | 1.56 | 1.36 | 1.82 | 0.0488 | * | Control vs WT-pRBC | 0.0571 |  |
|  |  | TKO-pRBC | 2.06 | 1.88 | 2.41 |  |  | Control vs TKO-pRBC | 0.1143 |  |
|  |  | WT-pRBC | 3.09 | 2.39 | 4.03 |  |  | WT-pRBC vs TKO-pRBC | 0.2000 |  |
| ALT | D0Tf | Control | 1.09 | 1.04 | 1.10 | 0.0231 | * | Control vs WT-pRBC | 0.0286 | * |
|  |  | TKO-pRBC | 1.35 | 1.28 | 1.50 |  |  | Control vs TKO-pRBC | 0.0286 | * |
|  |  | WT-pRBC | 1.99 | 1.32 | 2.65 |  |  | WT-pRBC vs TKO-pRBC | 0.6857 |  |
| TBIL | D0Tf | Control | 0.92 | 0.89 | 1.00 | 0.0345 | * | Control vs WT-pRBC | 0.0571 |  |
|  |  | TKO-pRBC | 1.63 | 1.60 | 2.06 |  |  | Control vs TKO-pRBC | 0.0286 | * |
|  |  | WT-pRBC | 1.97 | 1.34 | 2.57 |  |  | WT-pRBC vs TKO-pRBC | 0.6857 |  |
|  | D1 | Control | 0.91 | 0.82 | 0.93 | 0.0183 | * | Control vs WT-pRBC | 0.0286 | * |
|  |  | TKO-pRBC | 2.17 | 1.91 | 2.43 |  |  | Control vs TKO-pRBC | 0.0286 | * |
|  |  | WT-pRBC | 4.04 | 2.41 | 5.30 |  |  | WT-pRBC vs TKO-pRBC | 0.3429 |  |
| A/G | D3 | Control | 0.95 | 0.94 | 0.97 | 0.0210 | * | Control vs WT-pRBC | 0.4857 |  |
|  |  | TKO-pRBC | 1.09 | 1.04 | 1.11 |  |  | Control vs TKO-pRBC | 0.0286 | * |
|  |  | WT-pRBC | 0.94 | 0.92 | 0.95 |  |  | WT-pRBC vs TKO-pRBC | 0.0286 | * |
| BUN | D1 | Control | 0.92 | 0.84 | 0.96 | 0.0264 | * | Control vs WT-pRBC | 0.0571 |  |
|  |  | TKO-pRBC | 0.79 | 0.72 | 0.85 |  |  | Control vs TKO-pRBC | 0.3429 |  |
|  |  | WT-pRBC | 1.16 | 1.08 | 1.20 |  |  | WT-pRBC vs TKO-pRBC | 0.0286 | * |
|  | D7 | Control | 1.03 | 1.03 | 1.06 | 0.0244 | * | Control vs WT-pRBC | 0.0286 | * |
|  |  | TKO-pRBC | 0.79 | 0.74 | 0.85 |  |  | Control vs TKO-pRBC | 0.0286 | * |
|  |  | WT-pRBC | 0.84 | 0.73 | 0.95 |  |  | WT-pRBC vs TKO-pRBC | 0.8857 |  |
| GGT | D14 | Control | 0.94 | 0.89 | 0.99 | 0.0296 | * | Control vs WT-pRBC | 0.2000 |  |
|  |  | TKO-pRBC | 0.79 | 0.78 | 0.80 |  |  | Control vs TKO-pRBC | 0.0286 | * |
|  |  | WT-pRBC | 0.84 | 0.82 | 0.89 |  |  | WT-pRBC vs TKO-pRBC | 0.1143 |  |
|  | D21 | Control | 0.95 | 0.94 | 0.96 | 0.0366 | * | Control vs WT-pRBC | 0.3429 |  |
|  |  | TKO-pRBC | 0.83 | 0.81 | 0.85 |  |  | Control vs TKO-pRBC | 0.0286 | * |
|  |  | WT-pRBC | 0.90 | 0.87 | 0.95 |  |  | WT-pRBC vs TKO-pRBC | 0.1143 |  |
| TG | D0Bl | Control | 1.30 | 1.28 | 1.31 | 0.0366 | * | Control vs WT-pRBC | 0.3429 |  |
|  |  | TKO-pRBC | 1.07 | 1.02 | 1.12 |  |  | Control vs TKO-pRBC | 0.0286 | * |
|  |  | WT-pRBC | 1.17 | 1.15 | 1.35 |  |  | WT-pRBC vs TKO-pRBC | 0.1143 |  |
| PL | D7 | Control | 0.99 | 0.95 | 1.01 | 0.0231 | * | Control vs WT-pRBC | 0.1143 |  |
|  |  | TKO-pRBC | 1.07 | 1.02 | 1.15 |  |  | Control vs TKO-pRBC | 0.1143 |  |
|  |  | WT-pRBC | 0.83 | 0.77 | 0.87 |  |  | WT-pRBC vs TKO-pRBC | 0.0286 | * |
| TCHO | D7 | Control | 0.98 | 0.94 | 1.04 | 0.0097 | ** | Control vs WT-pRBC | 0.0286 | * |
|  |  | TKO-pRBC | 1.11 | 1.07 | 1.17 |  |  | Control vs TKO-pRBC | 0.0571 |  |
|  |  | WT-pRBC | 0.84 | 0.82 | 0.87 |  |  | WT-pRBC vs TKO-pRBC | 0.0286 | * |
| Ca | D3 | Control | 0.97 | 0.96 | 0.99 | 0.0244 | * | Control vs WT-pRBC | 0.0286 | * |
|  |  | TKO-pRBC | 0.96 | 0.95 | 0.97 |  |  | Control vs TKO-pRBC | 0.8857 |  |
|  |  | WT-pRBC | 0.91 | 0.88 | 0.93 |  |  | WT-pRBC vs TKO-pRBC | 0.0286 | * |
|  | D5 | Control | 0.96 | 0.95 | 0.98 | 0.0488 | * | Control vs WT-pRBC | 0.0286 | * |
|  |  | TKO-pRBC | 0.94 | 0.93 | 0.95 |  |  | Control vs TKO-pRBC | 0.2000 |  |
|  |  | WT-pRBC | 0.93 | 0.91 | 0.93 |  |  | WT-pRBC vs TKO-pRBC | 0.3429 |  |
|  | D7 | Control | 0.98 | 0.97 | 1.00 | 0.0125 | * | Control vs WT-pRBC | 0.0286 | * |
|  |  | TKO-pRBC | 0.95 | 0.95 | 0.96 |  |  | Control vs TKO-pRBC | 0.1143 |  |
|  |  | WT-pRBC | 0.93 | 0.92 | 0.94 |  |  | WT-pRBC vs TKO-pRBC | 0.0286 | * |
| GLU | D5 | Control | 0.86 | 0.76 | 1.08 | 0.0488 | * | Control vs WT-pRBC | 0.3429 |  |
|  |  | TKO-pRBC | 0.66 | 0.64 | 0.70 |  |  | Control vs TKO-pRBC | 0.2000 |  |
|  |  | WT-pRBC | 1.08 | 0.93 | 1.30 |  |  | WT-pRBC vs TKO-pRBC | 0.0286 | * |
| Iron | D1 | Control | 0.86 | 0.85 | 0.92 | 0.0244 | * | Control vs WT-pRBC | 0.0286 | * |
|  |  | TKO-pRBC | 1.85 | 1.44 | 2.46 |  |  | Control vs TKO-pRBC | 0.0286 | * |
|  |  | WT-pRBC | 2.00 | 1.73 | 2.14 |  |  | WT-pRBC vs TKO-pRBC | 0.8857 |  |
|  | D5 | Control | 0.48 | 0.39 | 0.59 | 0.0173 | * | Control vs WT-pRBC | 0.0571 |  |
|  |  | TKO-pRBC | 1.26 | 1.04 | 1.42 |  |  | Control vs TKO-pRBC | 0.0286 | * |
|  |  | WT-pRBC | 0.81 | 0.72 | 0.87 |  |  | WT-pRBC vs TKO-pRBC | 0.1143 |  |
|  | D14 | Control | 0.39 | 0.34 | 0.44 | 0.0125 | * | Control vs WT-pRBC | 0.1143 |  |
|  |  | TKO-pRBC | 0.55 | 0.49 | 0.64 |  |  | Control vs TKO-pRBC | 0.0286 | * |
|  |  | WT-pRBC | 0.45 | 0.43 | 0.46 |  |  | WT-pRBC vs TKO-pRBC | 0.0286 | * |
| TS | D1 | Control | 0.94 | 0.92 | 1.01 | 0.0244 | * | Control vs WT-pRBC | 0.0286 | * |
|  |  | TKO-pRBC | 1.94 | 1.48 | 2.54 |  |  | Control vs TKO-pRBC | 0.0286 | * |
|  |  | WT-pRBC | 2.11 | 1.83 | 2.25 |  |  | WT-pRBC vs TKO-pRBC | 0.8857 |  |
|  | D5 | Control | 0.45 | 0.38 | 0.52 | 0.0173 | * | Control vs WT-pRBC | 0.0571 |  |
|  |  | TKO-pRBC | 1.19 | 0.99 | 1.34 |  |  | Control vs TKO-pRBC | 0.0286 | * |
|  |  | WT-pRBC | 0.73 | 0.67 | 0.80 |  |  | WT-pRBC vs TKO-pRBC | 0.1143 |  |
| TIBC | D0Tf | Control | 0.90 | 0.89 | 0.92 | 0.0210 | * | Control vs WT-pRBC | 0.0286 | * |
|  |  | TKO-pRBC | 0.98 | 0.97 | 1.04 |  |  | Control vs TKO-pRBC | 0.0286 | * |
|  |  | WT-pRBC | 1.05 | 1.00 | 1.10 |  |  | WT-pRBC vs TKO-pRBC | 0.4857 |  |
| UIBC | D1 | Control | 0.96 | 0.90 | 1.01 | 0.0308 | * | Control vs WT-pRBC | 0.0286 | * |
|  |  | TKO-pRBC | 0.52 | 0.40 | 0.62 |  |  | Control vs TKO-pRBC | 0.0571 |  |
|  |  | WT-pRBC | 0.25 | 0.10 | 0.47 |  |  | WT-pRBC vs TKO-pRBC | 0.4857 |  |
|  | D5 | Control | 1.57 | 1.55 | 1.68 | 0.0125 | * | Control vs WT-pRBC | 0.0286 | * |
|  |  | TKO-pRBC | 1.01 | 0.96 | 1.09 |  |  | Control vs TKO-pRBC | 0.0286 | * |
|  |  | WT-pRBC | 1.24 | 1.15 | 1.32 |  |  | WT-pRBC vs TKO-pRBC | 0.1143 |  |

AST, aspartate aminotransferase; ALT, alanine aminotransferase; CK, creatine phosphokinase; GGT, gamma glutamyl transpeptidase; TP, total protein; ALB, albumin; A/G, albumin/globulin ratio; CREA, creatinine; TBIL, total bilirubin; TG, triglyceride; PL, phospholipids; TCHO, total cholesterol; Ca, calcium; TIBC, total iron-binding capacity; TS, transferrin saturation; UIBC, unsaturated iron-binding capacity.; K-W, the Kruskal–Wallis test; M-W U, the Mann–Whitney U test; *, *P* < 0.05; **, *P* < 0.01.

Supplemental Table 4. Median ratio or difference values and interquartile ranges of parameters that differed significantly among the first and second transfusion groups

| Parameter | Time | Transfusion | median | Q1 | Q3 | M-W U *P*-value |  |
| --- | --- | --- | --- | --- | --- | --- | --- |
| RBC | D1 | First | 0.9 | 0.9 | 1.1 | 0.0244 | * |
|  |  | Second | 0.7 | 0.7 | 0.8 |  |  |
| HGB | D1 | First | 0.9 | 0.8 | 1.0 | 0.0485 | * |
|  |  | Second | 0.8 | 0.7 | 0.8 |  |  |
|  | D14 | First | 0.9 | 0.9 | 1.0 | 0.0398 | * |
|  |  | Second | 0.9 | 0.9 | 0.9 |  |  |
| RETA | D1 | First | 1.2 | 1.1 | 1.2 | 0.0121 | * |
|  |  | Second | 1.7 | 1.6 | 1.8 |  |  |
|  | D3 | First | 1.7 | 1.6 | 2.0 | 0.0139 | * |
|  |  | Second | 3.5 | 3.5 | 4.4 |  |  |
|  | D14 | First | 2.5 | 2.3 | 3.2 | 0.0121 | * |
|  |  | Second | 6.5 | 5.3 | 7.0 |  |  |
|  | D21 | First | 1.3 | 1.1 | 1.5 | 0.0141 | * |
|  |  | Second | 3.0 | 2.7 | 3.8 |  |  |
| TBIL | D1 | First | 2.4 | 1.9 | 3.4 | 0.0121 | * |
|  |  | Second | 15.9 | 13.3 | 16.2 |  |  |
| IL6 | D1 | First | 2.2 | -0.2 | 6.6 | 0.0121 | * |
|  |  | Second | 28.2 | 19.3 | 35.7 |  |  |
|  | D5 | First | -7.6 | -13.2 | -2.2 | 0.0485 | * |
|  |  | Second | 3.4 | 0.1 | 4.2 |  |  |
|  | D14 | First | -8.2 | -13.5 | -0.9 | 0.0121 | * |
|  |  | Second | -0.3 | -1.8 | 0.6 |  |  |
| MCP1 | D5 | First | -31.3 | -54.5 | -13.9 | 0.0485 | * |
|  |  | Second | 16.1 | 8.5 | 36.7 |  |  |
| IFNγ | D0Tf | First | 0.3 | 0.2 | 0.5 | 0.0141 | * |
|  |  | Second | 11.3 | 7.4 | 13.6 |  |  |
|  | D3 | First | 6.0 | 4.0 | 7.9 | 0.0121 | * |
|  |  | Second | 0.6 | 0.2 | 0.7 |  |  |
| C3a | D0Tf | First | 0.1 | 0.1 | 0.2 | 0.0141 | * |
|  |  | Second | 1.0 | 0.5 | 1.5 |  |  |
| Factor Bb | D0Tf | First | 7.6 | 6.2 | 9.8 | 0.0121 | * |
|  |  | Second | 22.5 | 15.3 | 24.9 |  |  |
| Anti-pig cell IgM | D1 | First | -12.5 | -40.8 | 5.4 | 0.0242 | * |
|  |  | Second | -107.0 | -168.6 | -46.9 |  |  |
|  | D3 | First | 5.8 | -19.8 | 36.9 | 0.0485 | * |
|  |  | Second | -83.5 | -151.8 | -11.4 |  |  |
|  | D5 | First | 265.5 | 193.8 | 334.8 | 0.0121 | * |
|  |  | Second | -17.0 | -51.5 | 22.1 |  |  |
|  | D7 | First | 554.5 | 514.3 | 617.3 | 0.0121 | * |
|  |  | Second | -25.0 | -68.1 | 12.5 |  |  |
| Anti-pig cell IgG | D0Tf | First | -13.0 | -21.4 | -6.4 | 0.0121 | * |
|  |  | Second | -349.8 | -702.6 | -87.1 |  |  |
|  | D1 | First | -2.0 | -21.3 | 7.8 | 0.0121 | * |
|  |  | Second | -308.5 | -449.5 | -201.6 |  |  |
|  | D5 | First | 12.3 | -10.1 | 22.4 | 0.0141 | * |
|  |  | Second | 1145.5 | 486.0 | 1820.6 |  |  |
|  | D7 | First | 231.5 | 157.5 | 301.4 | 0.0121 | * |
|  |  | Second | 1453.0 | 844.0 | 1915.9 |  |  |
|  | D14 | First | 698.8 | 607.0 | 952.3 | 0.0121 | * |
|  |  | Second | 1585.3 | 1165.8 | 1947.6 |  |  |
|  | D21 | First | 947.0 | 670.4 | 1186.3 | 0.0121 | * |
|  |  | Second | 1527.0 | 1001.8 | 1727.4 |  |  |
| Aagglutination titer (fold difference) | D1 | First | 0.0 | -2.0 | 0.0 | 0.0301 | * |
|  |  | Second | -3.0 | -4.0 | -1.5 |  |  |
|  | D7 | First | 8.0 | 7.8 | 8.3 | 0.0209 | * |
|  |  | Second | 4.0 | 3.0 | 4.5 |  |  |
|  | D14 | First | 7.5 | 6.0 | 8.3 | 0.0176 | * |
|  |  | Second | 4.5 | 3.0 | 5.3 |  |  |

RBC, red blood cell; HGB, hemoglobin; RETA, reticulocyte; TBIL, total bilirubin; IL, interleukin; MCP-1, monocyte chemoattractant protein-1; IFN, interferon; M-W U, the Mann–Whitney U test; *, *P* < 0.05.

Supplemental Table 5. Reagents used in this study and the corresponding manufacturer information

| Item | Product information (Company) | Clone name |
| --- | --- | --- |
| Factor Bb | MicroVue™ Bb plus EIA (Quidel) | Not specified |
| C3a | BD OptEIA™ Human C3a ELISA Kit (BD Biosciences) | Not specified |
| C4a | BD OptEIA™ Human C4a ELISA Kit (BD Biosciences) | Not specified |
| Cytokines* | BD Cytometric Bead Assay (CBA) Human Inflammatory Cytokines Kit | Not specified |
| Interleukin-1 alpha | Monkey Interleukin-1 Alpha (IL1A) ELISA Kit (Cusabio) | Not specified |
| Interleukin-15 | Monkey Interleukin 15 (IL-15) ELISA Kit (Cusabio) | Not specified |
| MCP-1 | Monkey MCP1 SimpleStep ELISA® Kit (Abcam) | Not specified |
| Interferon-γ | ELISA Pro: Monkey IFN-γ (Mabtech) | Not specified |

Cytokines include interleukin-8 (IL-8), interleukin-1β (IL-1β), interleukin-6 (IL-6), interleukin-10 (IL-10), tumor necrosis factor (TNF), and interleukin-12p70 (IL-12p70)
